# Element Geochemical Analysis of the Contribution of Aeolian Sand to Suspended Sediment in Desert Stream Flash Floods

**DOI:** 10.1155/2014/620610

**Published:** 2014-06-26

**Authors:** Xiaopeng Jia, Haibing Wang

**Affiliations:** Key Laboratory of Desert and Desertification, Cold and Arid Regions Environmental and Engineering Research Institute, Chinese Academy of Sciences, Donggang West Road 260, Lanzhou, Gansu Province 730000, China

## Abstract

The interaction of wind and water in semiarid and arid areas usually leads to low-frequency flash flood events in desert rivers, which have adverse effects on river systems and ecology. In arid zones, many aeolian dune-fields terminate in stream channels and deliver aeolian sand to the channels. Although aeolian processes are common to many desert rivers, whether the aeolian processes contribute to fluvial sediment loss is still unknown. Here, we identified the aeolian-fluvial cycling process responsible for the high rate of suspended sediment transport in the Sudalaer desert stream in the Ordos plateau of China. On the basis of element geochemistry data analysis, we found that aeolian sand was similar to suspended sediment in element composition, which suggests that aeolian sand contributes to suspended sediment in flash floods. Scatter plots of some elements further confirm that aeolian sand is the major source of the suspended sediment. Factor analysis and the relation between some elements and suspended sediment concentration prove that the greater the aeolian process, the higher the suspended sediment concentration and the greater the contribution of aeolian sand to suspended sediment yield. We conclude that aeolian sand is the greatest contributor to flash floods in the Sudalaer desert stream.

## 1. Introduction

Wind and water are two basic geomorphic erosion processes. Their interaction in desert rivers in semiarid and arid areas usually leads to low-frequency flash flood events [1–10], which have adverse effects on river systems and ecology. As a result of the availability of erodible material on poorly vegetated slopes, desert rivers transport larger quantities of sediment during flood events and produce higher suspended sediment concentrations than rivers in humid regions [1, 3, 4, 11–13]. Desert rivers are significant agents of erosion, deposition, and landform development [14, 15].

A distinctive characteristic of some desert rivers is that they interact with adjacent aeolian dune-fields [8, 9]. Some evidence suggests that many active aeolian dune-fields terminate in desert rivers and aeolian sand may even truncate the channels of some ephemeral desert streams [16–21], for example, the Strzelecki desert at the Cooper Creek River in South Australia [16], the Namib desert at the Kuiseb channel in Africa [17, 18], the Simpson desert at the Finke River in Australia [19], and the Ulan Buh desert at the Yellow River in China [21]. Although the nature of these interactions varies greatly depending on local circumstances, aeolian processes are common to many desert rivers. However, whether aeolian sand affects flash floods in desert rivers is still unknown, because wind and water erosion are traditionally studied separately and high infiltration rates of rainfall on sand dunes seem to preclude runoff in desert regions, and thus aeolian sand does not influence fluvial processes. But on the basis of field observations in some desert streams, active aeolian sand dunes have been found to move downwind and deposit sand in the channels during the windy season; this sand may be transported downstream in the form of suspended sediment by floods in the rainy season. So, a fill-scour model for aeolian sand could have great effect on the fluvial processes of flash floods, but there is a lack of suitable data to support this theory.

We chose Sudalaer ephemeral desert stream on the Ordos desert plateau in northern China as a case study to reveal the effect of aeolian sand on suspended sediment during flash floods in desert streams.

## 2. Study Area

**Figure 1:**
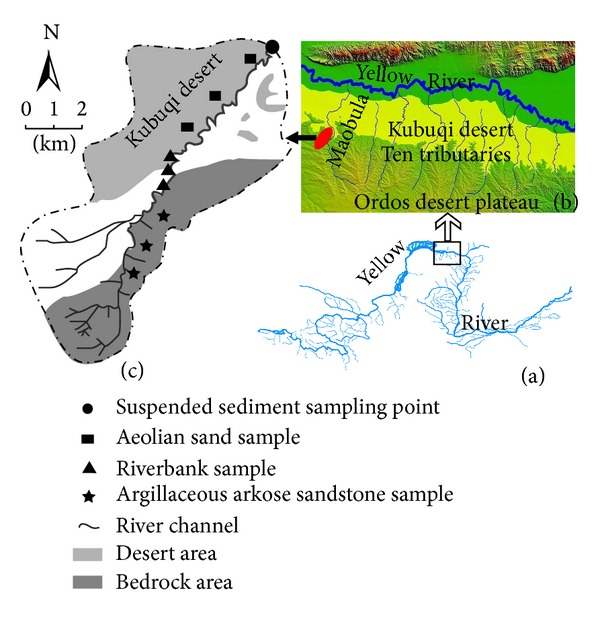
The location of the Sudalaer desert stream and sampling site. (a) shows the Yellow River; (b) locates the ten tributaries that originate in the high Ordos desert plateau, flow across the Kubuqi desert from south to north, and transport sediment to the Yellow River; (c) shows the Sudalaer desert stream and the sampling site.

The Sudalaer stream lies on the southern margin of the Kubuqi desert in the Ordos plateau of China. It is a tributary of Maobula desert stream, which originates in the high Ordos desert plateau, flows across the Kubuqi desert from south to north, and transports sediment to the Yellow River during the rainy season (Figure 1). Average annual precipitation in this region is 150–200 mm, most of which falls as heavy rain in July and August, resulting in flood events once every three years on average for the Sudalaer desert stream. In our study period from 2011 to 2012, five flood events occurred, of which four floods were surprisingly concentrated in July 2012.

**Figure 2:**
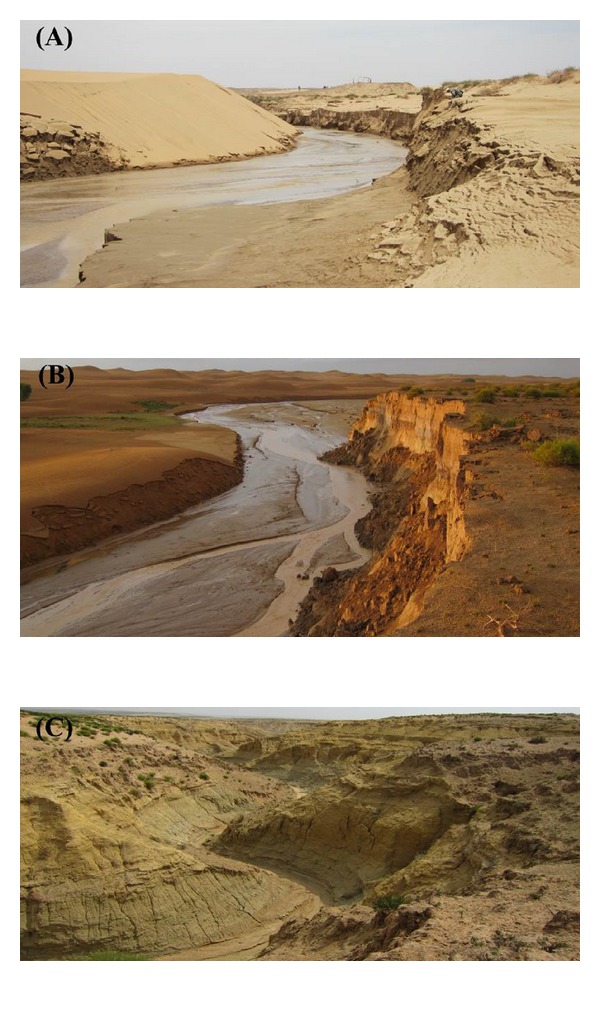
Surface characteristics of three reaches of the Sudalaer desert stream. (A)–(C) represent lower, middle, and upper reaches, respectively.

The 17 km Sudalaer desert stream has a gravel-bed channel, with a 59 km^2^ catchment area. The stream is divided into three sections according to spatial differences in the slope material and temporal differences in wind and water erosion forces. In the lower reach, the slopes are covered by aeolian sand, and aeolian processes dominate (Figure 2(A)). The 2–10 m high dunes, which migrate from the Kubuqi desert under the influence of northwesterly winds, are distributed on the left bank and terminate in the channel. The gravel bed in this reach is covered with 10–60 cm of aeolian sand in the windy season, and some channel sections are truncated by sand dunes. The middle reach is a transition zone, which is 10–30% covered with* Artemisia ordosica* Krasch shrubs and characterized by water erosion and weak wind erosion. The channel bank in this reach is mainly composed of loess and palaeoaeolian sand, with severe bank failures (Figure 2(B)). In the upper reach, exposed Cretaceous argillaceous arkose sandstones are widespread, and the channel is characterized by gullies and rills caused by severe water erosion (Figure 2(C)).

Coupled aeolian and fluvial processes are typical in the study region. During winter and spring, sand dunes from the Kubuqi desert migrate into the channel, leading to a narrowing or damming of the channel. Thunderstorms occur in summer and autumn, producing surface runoff and severe erosion, and some of the aeolian sand is carried downstream in the suspended sediments by floods.

## 3. Methods

The higher variability of sediment load transport and flow in desert rivers leads to great variability in sediment yields, making these yields difficult to predict [22]. In Sudalaer desert stream, the three different geomorphic units and erosion processes make it very difficult to estimate sediment yield per unit. Because geochemical properties of suspended sediments are retained during erosion, transport, and deposition, these properties can be used to infer sediment sources and transport routes [23]. Element geochemistry was used in this study to confirm whether aeolian sand affects suspended sediments in desert stream flash floods.

There were five flood events during our study period from 2011 to 2012, and suspended sediment samples from two flood events were analyzed (Table 1). The two flood events were the first floods in Sudalaer stream in 2011 and 2012 and are thus representative.

**Figure 3:**
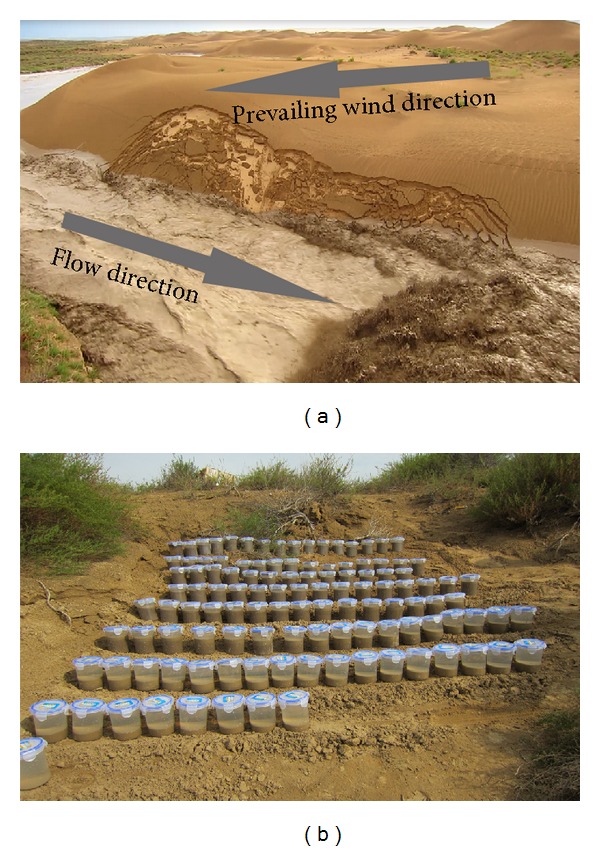
Flash flood event in the Sudalaer desert stream on August 23, 2011 (a), and suspended sediment samples are stored in plastic bottle (b).

Figure 3(a) shows a flash flood in the Sudalaer desert channel on August 23, 2011. Suspended sediment samples were collected within 50 cm of the water surface using a 480 mL or 550 mL bottle fixed on a 2.5 m long stainless steel rod. The sampling intervals were one minute, two minutes, three minutes, five minutes, and 10 minutes. Samples were stored in plastic bottles (Figure 3(b)) and brought to the laboratory. In the laboratory, they were dried at 100°C for 24 h and weighed to estimate the suspended sediment concentrations. Samples were sieved using the 1/3*ϕ* Udden-Wentworth grade scale [24, 25] to determine their size distribution.

**Figure 4:**
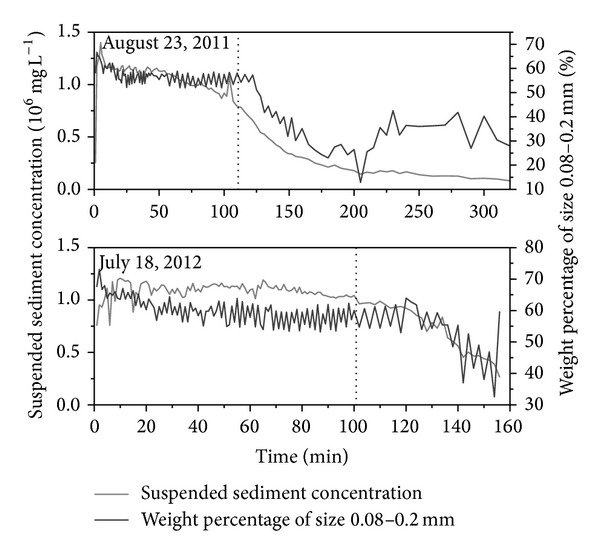
Changing suspended sediment concentration and size fraction of the suspended sediment during the two flood events in the Sudalaer desert stream.

In response to heavy rainfall, flows in the Sudalaer desert channel experienced very high sediment loads, and the 0.08–0.2 mm size fraction was the principal component (Figure 4). Element geochemistry analysis was focused on this size fraction. Aeolian sand, channel bank, and argillaceous arkose sandstone samples were washed and sieved to get the 0.08–0.20 mm size fraction to be compared with the suspended sediment.

A fully automated sequential wavelength-dispersive X-ray fluorescence (XRF) spectrometer (AXIOS, PANalytical B.V., Almelo, The Netherlands) equipped with a Super Sharp Tube for the Rh-anode was used for elemental analysis, with the following settings: 4.0 kW, 60 kV, 160 mA, and a 75 *μ* UHT Be end window. We used version 5 of the company's SuperQ software for the XRF analyses. The samples were crushed to less than 75 *μ*m using a multipurpose grinder and dried in an oven at 105°C; then 4 g of the dry powdered materials was pressed into a 32-mm-diameter pellet at 30-ton pressure using the pressed powder pellet technique. The briquettes were then stored in desiccators. After the XRF analyses were finished, the concentrations of the elements were calibrated using the Chinese National Standards for rock (GBW07103 and GBW07114 (GSR01 and GSR12), GBW07120 and GBW07122 (GSR13 and GSR15)), for soil (GBW07401 and GBW07408 (GSS01 and GSS8), GBW0743 and GBW07430 (GSS9 and GSS16)), and for water sediments (GBW07301a and GBW07318 (GSD01 and GSD14)). Concentrations of 28 elements and oxides were determined using the X-ray fluorescence spectrometer at the Key Laboratory of Desert and Desertification, Chinese Academy of Sciences. The manufacturer's specifications state that the analytical uncertainties (relative standard deviations) are less than ±5% for most elements and oxides under laboratory conditions.

Given the large amount of element data and the complex relationships among these data, multivariate statistical analysis methods, such as factor analysis and correlation analysis, have been widely used to interpret geochemical data for sediments [26–29]. These analytical methods can extract regularity from a variety of complex and messy data. For example, correlation analysis is a group of multivariate techniques whose primary purpose is to assemble objects based on the characteristics they possess and indicate patterns [27, 28], whereas factor analysis investigates intergrowth associations among variables and extracts useful information from mass data so the relative importance of these variables can be evaluated [26–29]. To better interpret relationships among these elements, factor and correlation analyses were performed for 16 main elements using the software SPSS v. 18.0 for Windows.

## 4. Results and Discussion

### 4.1. Element Contents of the Sediments

The average weight percentages of elements in samples are summarized in Table 2.

SiO_2_, Al_2_O_3_, CaO, MgO, Ti, Mn, Ba, P, Ce, Co, Zr, and Sr are the major chemical components, of which SiO_2_ shows a higher weight percentage than other major elements and oxides, and Ti shows a higher weight percentage than other trace elements (Table 2). Element contents were very similar for suspended sediments of the two flood events in Sudalaer stream. The lower standard deviations of these elements indicate less variability and that the majority of the suspended sediment came from a single source. Comparing element contents of suspended sediment samples and source areas, we found that most samples fall in the ranges of aeolian sand and channel bank material and out of the range of argillaceous arkose sandstone (Table 2). This indicates that aeolian sand from the Kubuqi desert and channel bank material composed of palaeoaeolian sand have a large effect on suspended sediments in flash floods of the Sudalaer desert stream. The standard deviations of most elements in argillaceous arkose sandstone samples are higher than those in aeolian sand and channel bank samples (Table 2), indicating that the geochemical data are relatively disperse; this is largely related to strong physical and chemical weathering in the upper reach of the Sudalaer desert stream.

**Figure 5:**
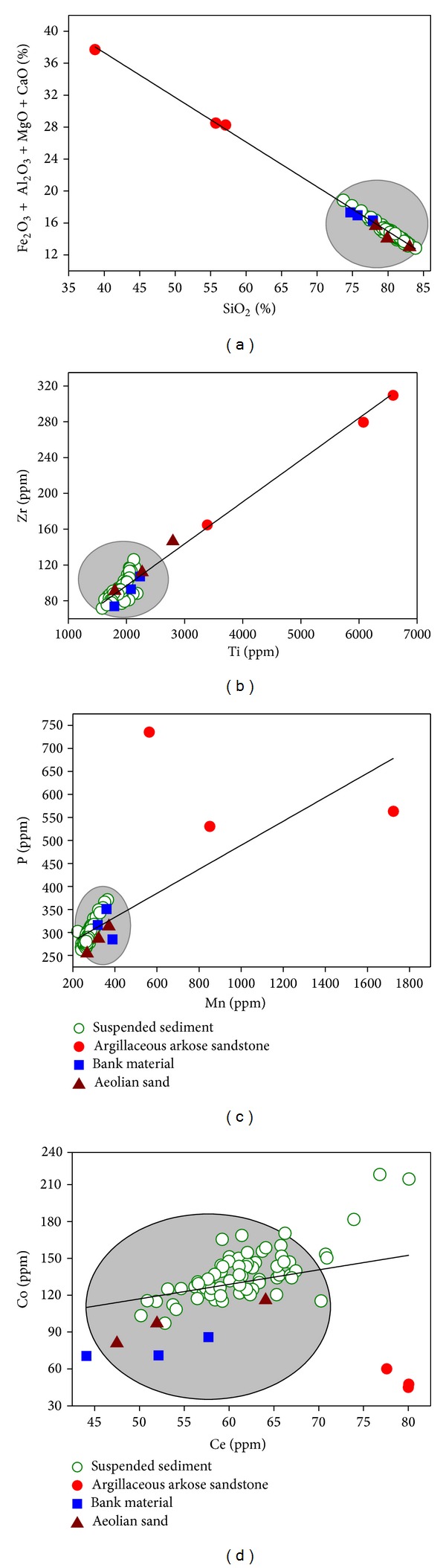
Scatter plots of some elements in samples collected in the Sudalaer basin.

Correlation analysis shows that in the suspended sediment, Si is negatively correlated with the major elements Al, Fe, Mg, and Ca and with the trace elements Mn, Ti, P, Sr, and Rb (Table 3), indicating that the properties of Si are different from those elements [30]. As a result of similar crystalline chemical properties, Mn^+2^ can be substituted for Fe^+2^, Mg^+2^, or Ca^+2^ [31] and Mn may thus be incorporated into Fe- or Mg-bearing minerals; as expected, there are high positive correlations among these elements (Table 3). Strontium may be enriched in Ca-bearing minerals [32]; statistically Sr shows a good positive correlation with Ca (Table 3). The high positive correlation of Ba : K indicates that the two elements have similar carriers, feldspar, and mica [33] (Table 3). The correlation coefficient between Co and Ce is 0.735; these two elements show very low positive or negative correlations with other elements (Table 3), all of which suggest Co and Ce have the same properties. Ti has a high concentration and shows a positive correlation with Mn, Zr, and Fe (Table 3), indicating enrichment of Ti-bearing minerals in suspended sediment. The high positive correlation among these elements indicates that they may have the same provenance [30]. Scatter plots of some elements in the samples further confirm the relationships among these elements and also indicate that the suspended sediments collected in Sudalaer stream are mainly from aeolian sand and channel banks (Figure 5). This not only suggests that there is little sediment available for erosion in the upper reach of the Sudalaer stream, while bank material and aeolian sand provide more available sediment, but also illustrates that vegetation growth on the channel bed and lower bank may play an important role in intercepting the coarse sediment from the upper reach [34].

### 4.2. Temporal Changes in Element Composition and *R*-Factor Model Analysis

**Figure 6:**
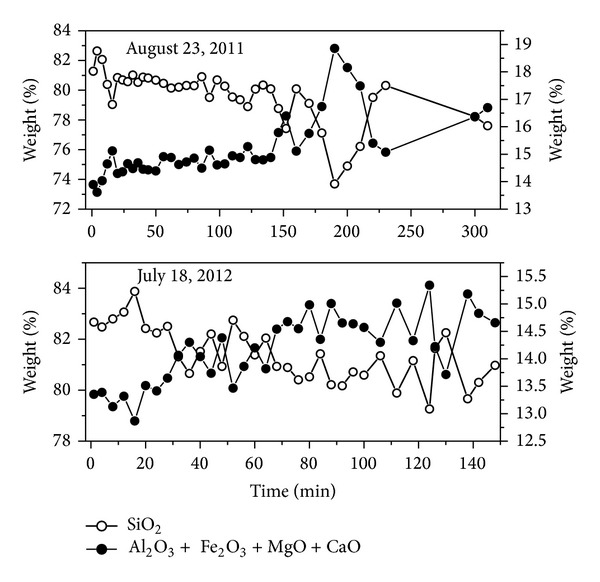
Temporal changes in elements Si and Al+Fe+Mg+Ca during the two flood events.

**Figure 7:**
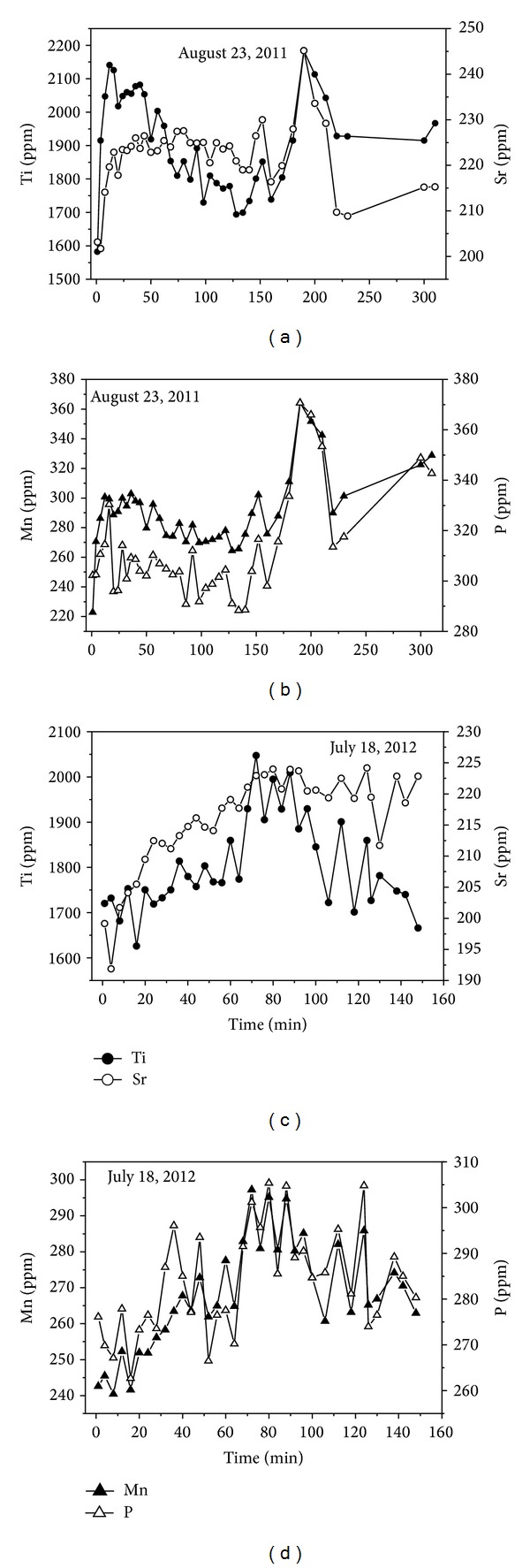
Temporal changes in elements Ti and Sr, Mn and P during the two flood events.

**Figure 8:**
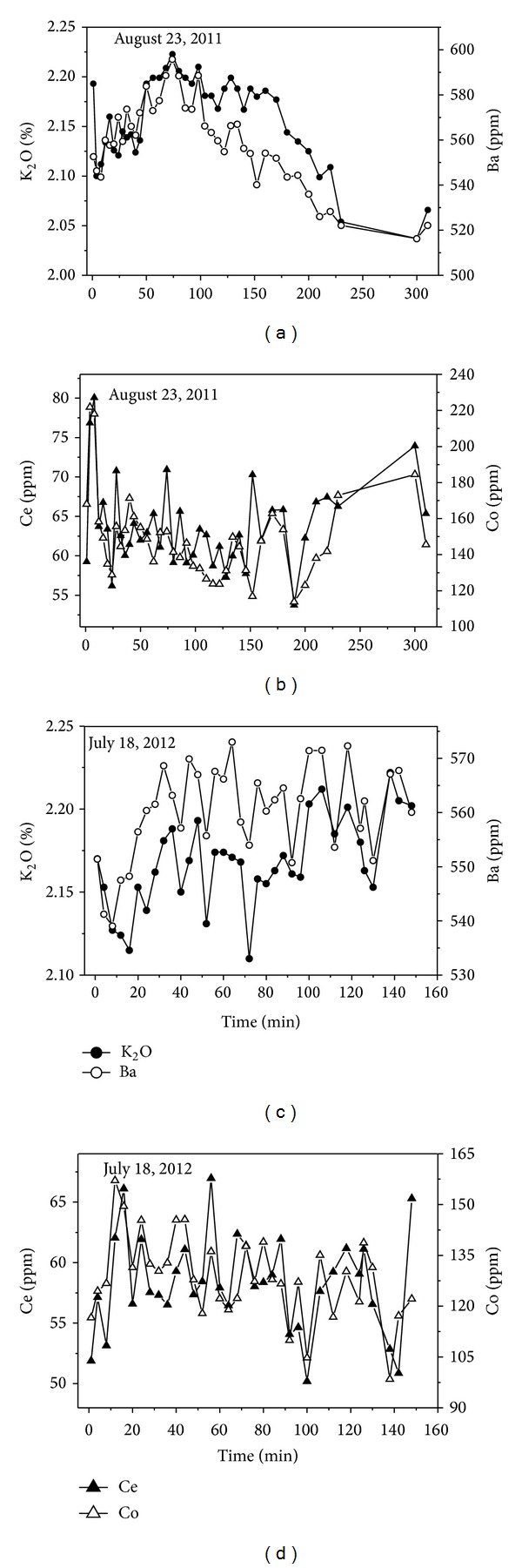
Temporal changes in elements K and Ba, Ce and Co during the two flood events.

Temporal changes in some elements are presented in Figures 6–8. Silica and other major elements (Al+Fe+Mg+Ca) take on an inverse trend with similar patterns during the two flood events (Figure 6). Negative correlation coefficients between Si and Al+Fe+Mg+Ca verified this trend (Table 3). The declining trend of Si and increasing trend of Al+Fe+Mg+Ca suggest that the ability of the two floods to transport sediment was limited by sediment availability in the aeolian sand-filled channel. Ti and Sr, Mn and P, Ce and Co, and K and Ba have positive correlation coefficients (Table 3), and they exhibit similar variation trends during a single flood event, but different patterns between the two flood events (Figures 7 and 8), which reflects the higher variability of suspended sediment transport in desert rivers [22] and higher mobility of these elements.

**Figure 9:**
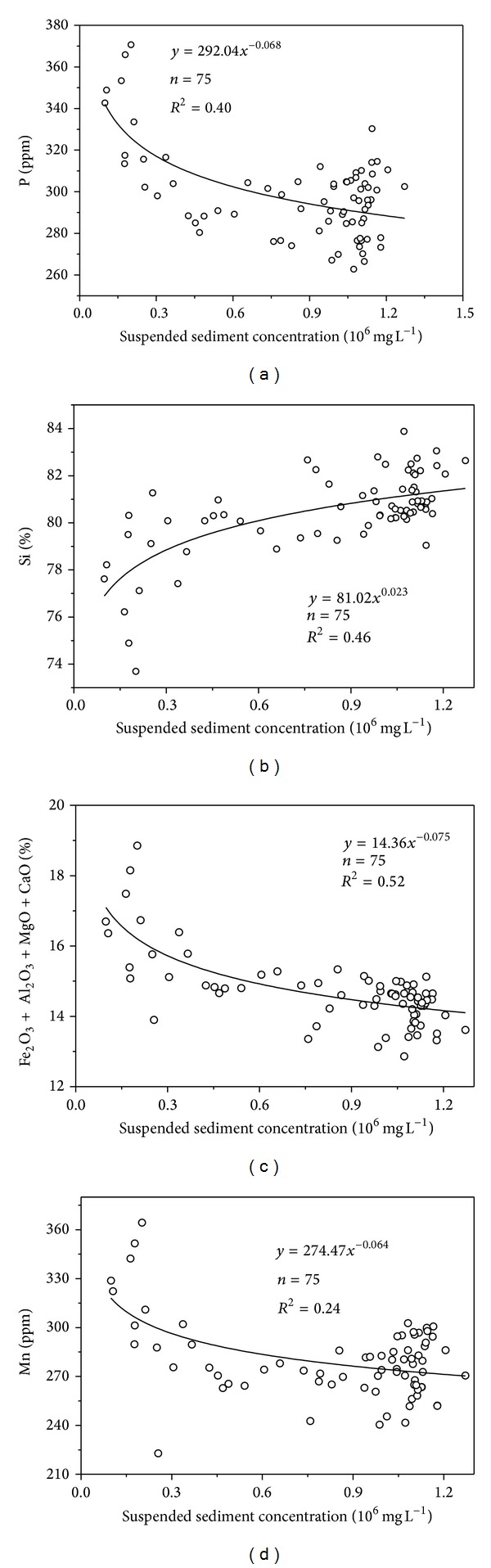
Relationships between suspended sediment concentration and some element contents.

The relationship between element content and suspended sediment concentration suggests that the higher the suspended sediment concentration, the higher the Si content but the lower the content of Mn, P, and Al+Fe+Mg+Ca (Figure 9). Because aeolian sand and bank material contain higher Si and lower Al, Fe, Mg, and Ca compared with argillaceous arkose sandstone (Table 2), these results indicate that aeolian sand has a greater effect on suspended sediment yield during higher suspended sediment concentration periods of the flood events. As the suspended sediment concentration declines with time (Figure 4) and sediment transport capacity is reduced, the contribution of aeolian sand to suspended sediment also decreases with time. Our principal components analysis showed that four principal components (PCs) with eigenvalues >1 could be extracted from the data and that these PCs explained 92.82% of the total variance (Table 4). If we consider only loadings with magnitudes greater than 0.60, PC1 included SiO_2_, CaO, MgO, Al_2_O_3_, Fe_2_O_3_, P, Mn, and Sr, where SiO_2_ had a negative value, while the other elements and oxides had positive values; this explains the inverse correlation between SiO_2_ and other elements (Table 3). For PC2, values that met this criterion were for Ba, K_2_O, and Na_2_O. For PC3, Zr and Ti met the criterion and for PC4, Co and Ce did so. For PC2, PC3, and PC4 the loading coefficients have positive values, which shows that these elements and oxides may have identical sources (i.e., they may come from the same or similar minerals).

**Figure 10:**
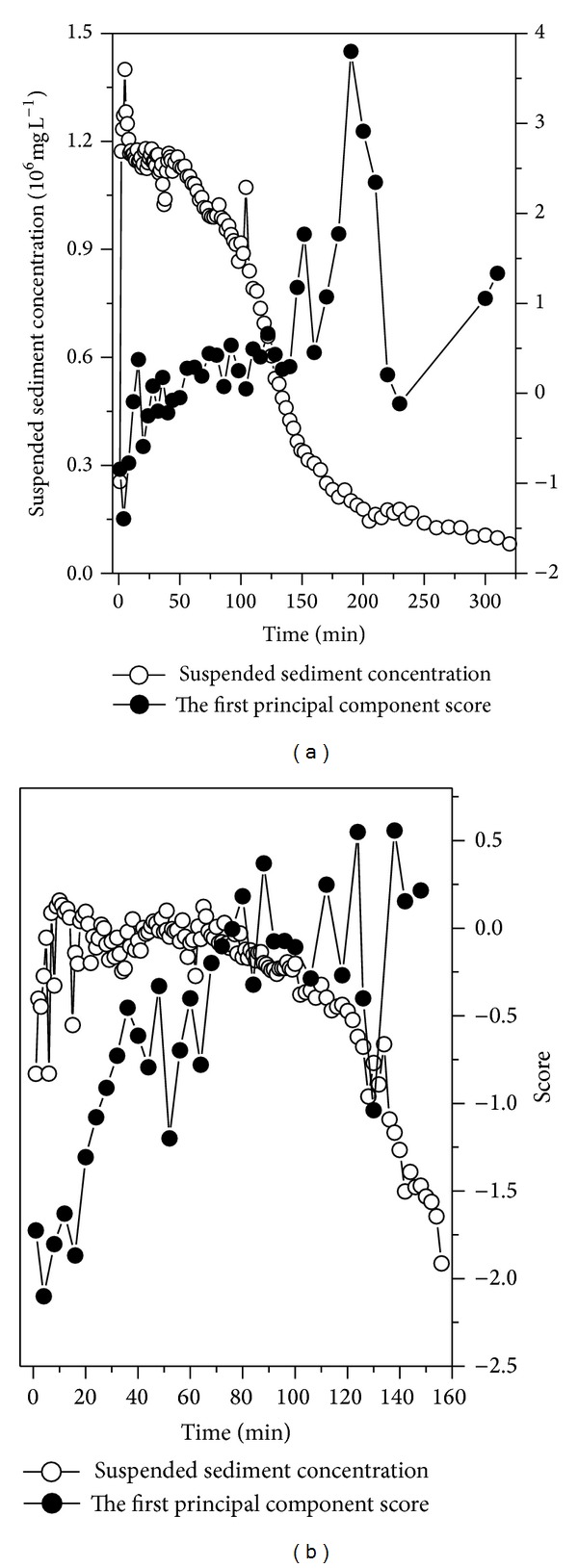
Temporal change in the first principal component score against the suspended sediment concentration.

According to the loading coefficient of PC1, if the first principal component score is negative, this indicates that Si occupies a considerable proportion. However, if the first principal component score is positive, this suggests that other elements account for a larger share. On the basis of the temporal change in the first principal component scores (Figure 10) and the element contents of the three source areas (Table 2), the effect of aeolian sand on suspended sediment in flash floods over time could be identified. The first principal component score has negative values at the initial high concentration stage (Figure 10), suggesting that Si has a higher proportion and other elements have lower proportions at this stage. This corresponds to the suspended sediment mainly coming from aeolian sand and channel bank material (Table 2). As suspended sediment concentration declines with time, the receding flow has little or no capacity to scour and carry away large amounts of aeolian sand. The first principal component score increases and exhibits positive values (Figure 10), illustrating that Si decreases as other elements increase with time; this corresponds to aeolian sand declining in the suspended sediment as total sediment load concentration decreases. This also suggests that with increased aeolian processes the higher the suspended sediment concentration, the greater the contribution of aeolian sand to suspended sediment yield. This suggests it is the aeolian sand that has the greatest contribution to sediment load in the flash floods in the Sudalaer desert stream. Earlier studies have shown that aeolian activity influences channel processes and landforms by controlling channel alignment or by ponding floodwaters [18, 35], but our results further point to aeolian sand as a major source of suspended sediment in some desert streams.

## 5. Conclusion

In arid zones, many aeolian dune-fields terminate in stream channels and deliver aeolian sand to the channels. Fluvial-aeolian interactions are distinctive aspects of some desert rivers. On the basis of element geochemistry data analysis, our results indicate that aeolian sand has significant effects on suspended sediment during flash floods in desert streams.

**Figure 11:**
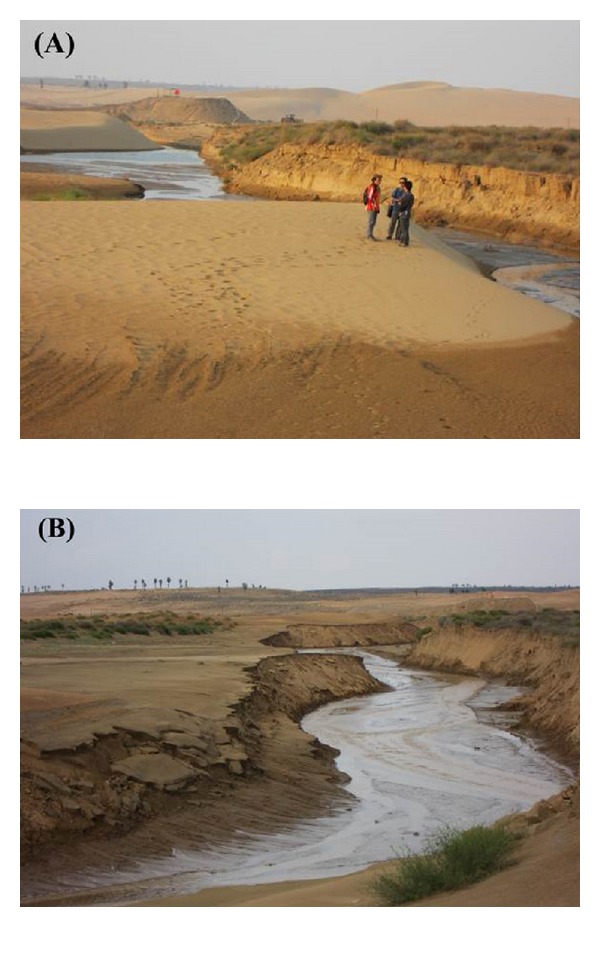
Graphic evidence of aeolian sand storage loss and lateral enlargement of the desert channel after the flood on August 23, 2011. (A) Preflood channel section in May 2011; (B) postflood channel section on August 26, 2011.

Our results also reveal a distinctive fill-scour model for some desert rivers. During the windy season, aeolian sand is blown into the river channels, leading to narrowing or damming of the channels. When rain storms come, flash floods form and these aeolian sands are transported downstream. The greater the aeolian process, the higher the suspended sediment concentration and the greater the contribution of aeolian sand to suspended sediment yield. The flood has a very high sediment load from the aeolian sand contribution, not only delivering larger amounts of sediment downstream but also widening the channel.  Figure 11 provides graphic evidence of aeolian dune erosion, leading to lateral enlargement of the channel.

## Figures and Tables

**Table 1:** Sampling number and duration of the two flood events in the Sudalaer desert stream.

Time	Duration of flood events (min)	Sample number of suspended sediment	Number of analyzed sample
August 23, 2011	320	44 (one-minute interval)	40
		30 (two-minute interval)	
		17 (three-minute interval)	
		17 (five-minute interval)	
		8 (10-minute interval)	

July 18, 2012	156	100 (one-minute interval)	35
		28 (two-minute interval)	

**Table 2:** Mean (±SD) concentration of elements in the suspended sediment and the three source areas.

Chemical element	Suspended sediment		Aeolian sand (*n* = 3)	Channel bank material (*n* = 3)	Argillaceous arkose sandstone (*n* = 3)
	August 23, 2011 (*n* = 40)	July 18, 2012 (*n* = 35)			
SiO_2_ (%)	79.64 ± 1.78	81.41 ± 1.07	80.41 ± 2.41	76.08 ± 1.62	50.52 ± 10.26
Al_2_O_3_ (%)	9.05 ± 0.43	8.73 ± 0.23	8.76 ± 0.40	9.15 ± 0.20	11.76 ± 1.47
Fe_2_O_3 _(%)	2.23 ± 0.29	1.98 ± 0.11	2.36 ± 0.41	2.32 ± 0.20	4.14 ± 0.77
MgO (%)	0.92 ± 0.19	0.78 ± 0.08	0.82 ± 0.16	1.15 ± 0.12	2.35 ± 0.70
CaO (%)	2.98 ± 0.26	2.69 ± 0.25	2.27 ± 0.37	4.24 ± 0.63	13.24 ± 8.07
Na_2_O (%)	2.48 ± 0.10	2.53 ± 0.05	2.62 ± 0.09	2.58 ± 0.03	2.63 ± 0.56
K_2_O (%)	2.16 ± 0.05	2.17 ± 0.03	2.03 ± 0.03	2.12 ± 0.02	2.10 ± 0.73
P (ppm)	311.59 ± 20.09	283.63 ± 11.39	284.45 ± 29.39	317.28 ± 32.91	609.72 ± 110.02
Ti (ppm)	1912.30 ± 145.63	1803.38 ± 102.16	2289.63 ± 499.22	2033.71 ± 225.19	5337.05 ± 1702.20
Mn (ppm)	290.80 ± 25.40	267.97 ± 15.16	320.61 ± 51.80	355.42 ± 35.26	1045.94 ± 604.47
Co (ppm)	148.35 ± 22.86	128.42 ± 12.37	99.20 ± 17.85	76.71 ± 8.87	51.29 ± 8.28
Ni (ppm)	26.39 ± 2.10	23.25 ± 1.19	20.19 ± 2.46	21.06 ± 1.98	18.29 ± 2.52
Rb (ppm)	73.84 ± 1.25	73.07 ± 0.90	67.99 ± 3.99	72.93 ± 1.66	53.70 ± 21.27
Sr (ppm)	222.06 ± 7.73	215.90 ± 7.82	174.94 ± 8.86	301.61 ± 101.20	457.83 ± 69.94
Zr (ppm)	93.18 ± 14.90	88.72 ± 6.83	117.49 ± 28.46	91.94 ± 17.09	254.40 ± 77.24
Ba (ppm)	558.45 ± 19.47	560.20 ± 8.65	507.77 ± 16.52	534.50 ± 4.31	710.23 ± 164.95
Ce (ppm)	63.86 ± 5.40	58.33 ± 4.01	54.51 ± 8.58	51.30 ± 6.85	79.22 ± 1.42
V (ppm)	37.39 ± 5.07	33.00 ± 2.33	42.11 ± 9.74	39.81 ± 3.93	73.39 ± 26.98
Cr (ppm)	33.37 ± 3.47	30.90 ± 2.11	42.29 ± 6.64	37.44 ± 4.29	39.36 ± 12.58
Cu (ppm)	13.47 ± 2.01	12.00 ± 1.02	8.77 ± 0.52	11.41 ± 1.26	11.97 ± 2.28
Ga (ppm)	11.29 ± 0.59	11.00 ± 0.45	10.23 ± 0.30	10.81 ± 0.64	13.67 ± 2.25
As (ppm)	6.82 ± 0.58	6.26 ± 0.38	7.00 ± 1.20	6.98 ± 1.08	1.27 ± 0.76
Y (ppm)	11.79 ± 0.62	11.34 ± 0.53	12.48 ± 1.09	12.32 ± 1.04	17.81 ± 2.21
Nb (ppm)	6.87 ± 0.47	6.49 ± 0.54	7.71 ± 0.96	6.73 ± 1.26	13.02 ± 2.99
Pb (ppm)	11.49 ± 1.09	11.96 ± 0.90	10.13 ± 2.05	13.15 ± 1.30	13.58 ± 3.26
Nd (ppm)	15.68 ± 2.77	14.08 ± 2.78	15.95 ± 1.29	15.40 ± 4.84	33.53 ± 5.91
Zn (ppm)	nd	nd	nd	8.31 ± 3.62	32.12 ± 13.75
La (ppm)	nd	nd	nd	nd	36.86 ± 4.60

Note: *n*: the sediment number; nd: no determination; SD: standard deviation.

**Table 3:** Values of Pearson's correlation coefficient between elements of the suspended sediment collected from flood events in the Sudalaer desert stream (*n* = 75).

	SiO_2_	Al_2_O_3_	Fe_2_O_3_	MgO	CaO	Na_2_O	K_2_O	Ti	Mn	Ba	P	Sr	Rb	Ce	Co	Zr
SiO_2_	1															
Al_2_O_3_	−0.954**	1														
Fe_2_O_3_	−0.907**	0.866**	1													
MgO	−0.968**	0.960**	0.958**	1												
CaO	−0.922**	0.825**	0.823**	0.855**	1											
Na_2_O	0.589**	−0.607**	−0.792**	−0.749**	−0.464**	1										
K_2_O	0.086	−0.020	−0.448**	−0.230*	0.018	0.560**	1									
Ti	−0.472**	0.303**	0.596**	0.426**	0.591**	−0.169	−0.434**	1								
Mn	−0.827**	0.728**	0.923**	0.834**	0.860**	−0.597**	−0.416**	0.820**	1							
Ba	0.299**	−0.336**	−0.547**	−0.461**	−0.064	0.694**	0.760**	−0.145	−0.338**	1						
P	−0.879**	0.823**	0.908**	0.868**	0.806**	−0.539**	−0.358**	0.686**	0.889**	−0.391**	1					
Sr	−0.705**	0.577**	0.550**	0.596**	0.893**	−0.221	0.245*	0.504**	0.679**	0.296**	0.550**	1				
Rb	−0.696**	0.714**	0.556**	0.685**	0.666**	−0.515**	0.191	−0.055	0.398**	−0.109	0.440**	0.595**	1			
Ce	−0.097	0.084	0.255*	0.147	0.112	−0.238*	−0.392**	0.282*	0.281*	−0.269*	0.380**	0.024	−0.043	1		
Co	0.147	−0.146	0.045	−0.102	−0.142	−0.011	−0.445**	0.223	0.082	−0.183	0.256*	−0.238*	−0.211	0.735**	1	
Zr	0.101	−0.296**	−0.021	−0.201	0.106	0.364**	−0.204	0.761**	0.297**	0.225	0.161	0.219	−0.452**	0.215	0.266*	1

**Correlation is significant at the 0.01 level (2-tailed).

*Correlation is significant at the 0.05 level (2-tailed).

**Table 4:** Principal components analysis for the 16 major elements in suspended sediments (*n* = 75). The table shows principal components (PCs) with eigenvalues >1.

Element or oxide	Principal components			
	PC1	PC2	PC3	PC4
SiO_2_	−0.969	0.140	−0.025	0.047
CaO	0.962	0.081	0.206	−0.030
MgO	0.936	−0.324	−0.056	−0.017
Al_2_O_3_	0.930	−0.163	−0.170	−0.031
Fe_2_O_3_	0.872	−0.459	0.133	0.075
P	0.834	−0.268	0.262	0.283
Mn	0.825	−0.291	0.447	0.093
Sr	0.815	0.398	0.266	−0.091
Rb	0.776	0.096	−0.459	−0.056
Ba	−0.165	0.927	0.123	−0.113
K_2_O	0.011	0.869	−0.309	−0.279
Na_2_O	−0.577	0.676	0.235	−0.052
Zr	−0.098	0.151	0.946	0.164
Ti	0.452	−0.166	0.857	0.138
Co	−0.135	−0.149	0.146	0.914
Ce	0.129	−0.147	0.096	0.907
